# The Clinical and Pathological Significance of Nectin-2 and DDX3 Expression in Pancreatic Ductal Adenocarcinomas

**DOI:** 10.1155/2015/379568

**Published:** 2015-07-30

**Authors:** Shan Liang, Zhulin Yang, Daiqiang Li, Xiongying Miao, Leping Yang, Qiong Zou, Yuan Yuan

**Affiliations:** ^1^Research Laboratory of Hepatobiliary Diseases, Second Xiangya Hospital, Central South University, Changsha, Hunan 410011, China; ^2^Department of Pathology, Second Xiangya Hospital, Central South University, Changsha, Hunan 410011, China; ^3^Department of Pathology, Third Xiangya Hospital, Central South University, Changsha, Hunan 410013, China

## Abstract

Pancreatic ductal adenocarcinoma (PDAC) is a highly malignant disease, but the genetic basis of PDAC is still unclear. In this study, Nectin-2 and DDX3 expression in 106 PDAC, 35 peritumoral tissues, 55 benign pancreatic lesions, and 13 normal pancreatic tissues were measured by immunohistochemical methods. Results showed that the percentage of positive Nectin-2 and DDX3 expression was significantly higher in PDAC tumors than in peritumoral tissues, benign pancreatic tissues, and normal pancreatic tissues (*P* < 0.01). The percentage of cases with positive Nectin-2 and DDX3 expression was significantly lower in PDAC patients without lymph node metastasis and invasion and having TNM stage I/II disease than in patients with lymph node metastasis, invasion, and TNM stage III/IV disease (*P* < 0.05 or *P* < 0.01). Positive DDX3 expression is associated with poor differentiation of PDAC. Kaplan-Meier survival analysis showed that positive Nectin-2 and DDX3 expression were significantly associated with survival in PDAC patients (*P* < 0.001). Cox multivariate analysis revealed that positive Nectin-2 and DDX3 expression were independent poor prognosis factors in PDAC patients. In conclusion, positive Nectin-2 and DDX3 expression are associated with the progression and poor prognosis in PDAC patients.

## 1. Introduction

Pancreatic ductal adenocarcinoma (PDAC) has one of the worst prognoses among cancers and is the fourth leading cause of cancer-related deaths worldwide [1]. In 2014, there were 39,590 pancreatic cancer deaths in the United States, and the annual deaths from PDAC continue to increase [2]. Despite the progress made in diagnostic technology, the survival statistics demonstrated that only 8% of new diagnoses are early-stage disease with resectable tumor, while most pancreatic cancers are diagnosed at advanced stages with metastasis [3]. A study from the National Cancer Database of USA revealed that only 52% of patients with early-stage disease underwent surgical resection [4]. Among these patients with local disease, the 5-year postsurgical survival rate was only 23% [2]. The survival rate in patients with unresectable tumors is lower than 1% [5]. Current radiotherapy, chemotherapy, and other therapeutic strategies exhibit limited impact on the prognosis of PDAC. This might be because of a lack of thorough understanding of the molecular and genetic bases of PDAC.

Poliovirus receptor-related 2 protein (PRR2), also called Nectin-2 or CD112, is a novel cell adhesion molecule of the immunoglobulin superfamily (IgSF) [6, 7]. Besides its functions as an adhesion molecule, Nectin-2 has other important biological functions, such as being a receptor of some viruses and binding to the surface CD226 receptor of NK and cytotoxic T lymphocytes (CTL) cells to activate NK cells and CTL killing activity. Nectin-2 is expressed on the surface of tumor cells, and its recognition by NK cells plays a role in antitumor immunity [8, 9]. Recently, Nectin-2 expression was found to be closely related to the tumorigenesis and prognosis of neuroblastoma [10], multiple myeloma [11], acute myeloid leukemia [12], and Juventus sarcoma [13]. Also, Nectin-2 is highly expressed in some epithelial cancers, such as breast cancer [14, 15], ovarian cancer [15], gallbladder cancer [16], and cervical cancer [17], and higher Nectin-2 expression was found in more malignant cancers with rapid progression and poor prognosis. The expression of Nectin-2 in PDAC has not been reported.

Human DDX3 is an ATP-dependent RNA helicase of the DEAD-box family and is widely expressed in a variety of tissues [18, 19]. DDX3 plays an important role in the regulation of gene expression, such as transcription, splicing, mRNA transport, and translation [20]. DDX3 is also involved in cell cycle regulation and apoptosis [21]. DDX3 functions as an ATP-dependent enzyme to promote p21WAF1/CIPI promoter activity and subsequent p21WAF1/CIPI gene expression [22], and DDX3 further inhibits tumor growth by serving as a tumor suppressor gene [23]. In recent years, DDX3 has been demonstrated to be associated with the occurrence of breast cancer [24], non-small cell lung cancer [25, 26], oral squamous cell carcinoma [27], and gallbladder cancer [16]. The expression and biological significance of DDX3 have not been reported in PDAC.

In this study, Nectin-2 and DDX3 expression in 106 PDAC, 35 peritumoral tissues, 55 benign pancreatic lesions, and 13 normal pancreatic tissues were measured by immunohistochemistry. The clinicopathological significance of Nectin-2 and DDX3 expression and their associations with the prognosis of PDAC were analyzed.

## 2. Materials and Methods

### 2.1. Clinical Data

106 PDAC tumors, 35 peritumoral tissues, 55 pancreatic tissues with benign lesions, and 13 normal pancreatic tissues were collected from January 2000 to December 2011. PDAC was diagnosed based on the recent US Armed Forces Institute of Pathology Fascicle on pancreatic neoplasms [28]. PDAC tumor tissues were collected before radiotherapy or chemotherapy. The peritumoral tissues were collected ≥2 cm from the edge of PDAC tumors. Forty-five PDAC cases were female, while 61 were male with an average age of 54.50 ± 11.53 years. Thirty-eight PDAC (35.9%) tumors were well differentiated, 35 (33.0%) were moderately differentiated, and 33 (31.1%) were poorly differentiated. Thirteen PDAC tumors had a maximum tumor size <3 cm, 68 had a maximum tumor size of 3 to 5 cm, and 25 had a maximum tumor size >5 cm. Twenty-nine cases had regional lymph node metastasis, and 64 had invasion to surrounding tissues and organs of the pancreas. Eleven, forty-two, thirty-seven, and sixteen patients had TNM clinical stages I, II, III, and IV disease, respectively. The survival information of 106 PDAC cases was collected by letters or telephone calls. The PDAC cases were followed up for 2 years. Among the 106 cases with PDAC, 77 patients survived shorter than 1 year and 29 patients survived longer than 1 year with a mean survival time of 9.44 ± 0.69 months. Twelve, ten, eight, and five peritumoral tissues were normal, mild dysplasia, moderate dysplasia, and severe dysplasia. The 55 benign pancreatic specimens were collected from 29 males and 26 females with 13 cases ≤45 years old and 42 cases >45 years old. Twenty, twenty, and fifteen cases with benign lesions had chronic pancreatitis, adenoma, and intraepithelial neoplasia, respectively. Ten, six, and four of the twenty patients with chronic pancreatitis had mild, moderate, and severe pancreatitis, respectively. Among the 20 adenomas, 15 were serous adenomas and 5 were mucinous adenomas, respectively. Four, three, and two adenomas had mild, moderate, and severe dysplasia, respectively. Among the 15 intraepithelial neoplasias, 6, 5, and 4 were grade I, grade II, and grade III intraepithelial neoplasia. Thirteen normal pancreatic tissues were collected from surgery of pancreatic adenoma. All tissues were treated with 4% formaldehyde and then paraffin-embedded.

### 2.2. Immunohistochemistry

The rabbit anti-human Nectin-2 and DDX3 antibodies were purchased from Abgent Company (California, USA). Nectin-2 and DDX3 were immunohistologically stained using EnVision detection kit (Dako Laboratories, California, USA). Briefly, 4 *µ*m sections were cut from paraffin-embedded tissues. After sections were deparaffinized and incubated with 3% H_2_O_2_, sections were incubated with Nectin-2 or DDX3 antibody for 1 hr at room temperature (RT). After washing with PBS, solution A was applied to the sections for 30 min at RT, followed by incubation with color development solution and hematoxylin counter staining. 400 cells from 10 random fields were counted. Cases were positive if ≥25% of cells were positive and negative if <25% of cells were positive [29]. Positive control biopsies were provided with the EnVision detection kit. 5% fetal bovine serum was used as negative control.

### 2.3. Statistical Analysis

Data were analyzed using SPSS15.0 statistical package. The relationships between Nectin-2 and DDX3 expression and histological or clinical factors were analyzed by *χ* ^2^ test or Fisher's exact test. Univariate survival analysis was conducted with Kaplan-Meier method (log-rank test). Multivariate analysis was performed with Cox proportional hazard model and the 95% confidence interval was calculated. *P* < 0.05 was considered statistically significant.

## 3. Results

### 3.1. Nectin-2 and DDX3 Expression in Normal, Benign, and Malignant Pancreatic Tissues

**Figure 1:**
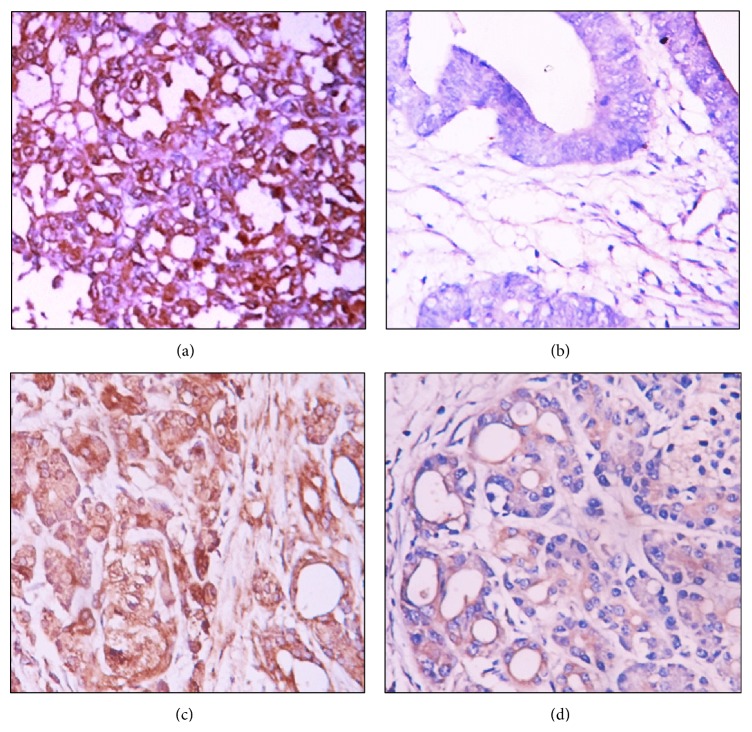
Nectin-2 expression in malignant and benign tissues. (a) Nectin-2 positive expression in poorly differentiated PDAC. (b) Nectin-2 negative expression in well differentiated PDAC. (c) Nectin-2 positive expression in peritumoral tissue. (d) Nectin-2 positive expression in adenoma. Magnification ×200. Cells with brown granules in the cytoplasm were identified as Nectin-2 positive.

**Figure 2:**
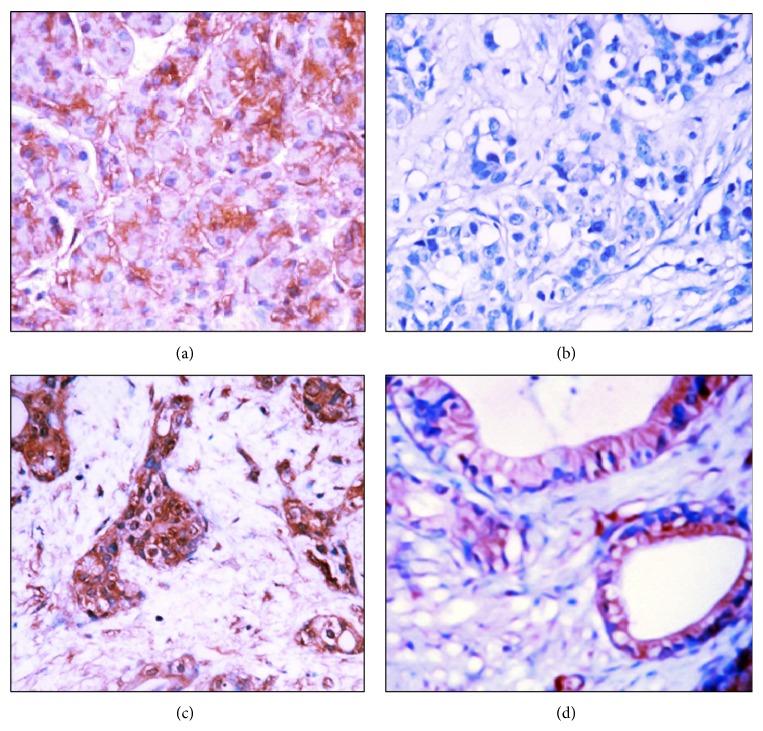
DDX3 expression in malignant and benign tissues. (a) DDX3 positive expression in poorly differentiated PDAC. (b) DDX3 negative expression in moderately differentiated PDAC. (c) DDX3 positive expression in intraepithelial neoplasia. (d) DDX3 positive expression in chronic pancreatitis tissue. Magnification ×200. Cells with brown granules in the cytoplasm were identified as DDX3 positive.

Positive Nectin-2 (Figure 1) and DDX3 (Figure 2) expression were mainly located in the cytoplasm. Among the 106 cases with PDAC, positive Nectin-2 and DDX3 expression were observed in 58 and 55 cases, respectively. Among the 35 peritumoral tissues, positive Nectin-2 and DDX3 expression were observed in 9 and 8 tissues, respectively. Among the 55 cases with benign pancreatic lesions, positive Nectin-2 and DDX3 expression were observed in 10 and 11 cases, respectively. Both Nectin-2 and DDX3 were negative in all 13 normal pancreatic tissues. Peritumoral tissues and benign lesions with positive Nectin-2 and DDX3 expression exhibited dysplasia or grade II or III intraepithelial neoplasia (Table 1). The percentage of positive Nectin-2 and DDX3 expression was significantly higher in PDAC tumors than in peritumoral tissues, benign lesions, and normal pancreatic tissues (*P* < 0.01 or *P* < 0.001). Among the 55 cases with benign pancreatic lesions, the percentages of positive Nectin-2 expression in chronic pancreatitis, adenomas, and intraepithelial neoplasia were 15.0%, 20.0%, and 20.0%, respectively. The percentages of positive DDX3 expression in chronic pancreatitis, adenoma, and intraepithelial neoplasia were 10.0%, 30.0%, and 20.0%, respectively. No significant differences in the percentage of positive Nectin-2 and DDX3 expression were observed between three types of benign lesions (*P* > 0.05).

### 3.2. Associations between Nectin-2 and DDX3 Expression and Clinicopathological Features of PDAC

The percentage of positive Nectin-2 and DDX3 expression was significantly higher in PDAC cases with poor differentiation, invasion to surrounding tissues and organs, lymph node metastasis, and TNM stages III and IV disease than in cases with well differentiated tumor, no invasion, no lymph node metastasis, and TNM stages I and II disease (*P* < 0.05 or *P* < 0.01) (Table 2). No significant differences were observed in patients with different age, sex, and tumor size (*P* > 0.05). Among the 58 cases with positive Nectin-2 expression, positive DDX3 expression was observed in 45 cases. Of the 45 cases with negative Nectin-2 expression, 38 cases were DDX3 negative, suggesting positive correlation between Nectin-2 and DDX3 expression in cases with PDAC (*P* = 0.000).

### 3.3. The Relationship between Clinicopathological Characteristics, Nectin-2 and DDX3 Expression, and Survival of PDAC Patients

**Figure 3:**
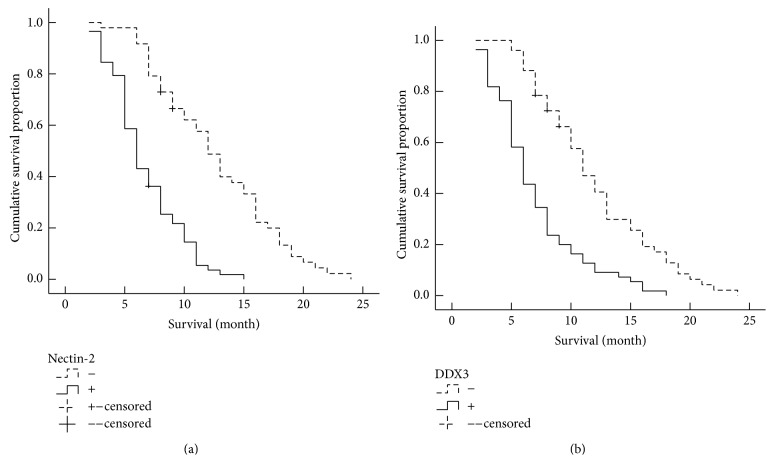
Nectin-2 and DDX3 expression and survival in patients with PDAC. (a) Kaplan-Meier plots of overall survival in patients with PDAC and with positive and negative Nectin-2 expression. (b) Kaplan-Meier plots of overall survival in patients with PDAC and with positive and negative DDX3 expression.

Kaplan-Meier survival analysis showed that poor differentiation, larger maximum tumor size, high TNM stage, lymph node metastasis and invasion, and positive Nectin-2 and DDX3 expression are significantly correlated with the shorter survival in PDAC cases (*P* < 0.05, *P* < 0.01, or *P* < 0.001). Patients with positive Nectin-2 and DDX3 expression survived significantly shorter than patients with negative Nectin-2 and DDX3 expression (*P* = 0.000) (Table 3, Figure 3). Cox multivariate analysis showed that differentiation, tumor size, TNM stage, lymph node metastasis, and invasion were independent prognostic factors and negatively correlated with survival. Positive Nectin-2 (Table 4) and DDX3 (Table 5) expression negatively correlated with survival, positively correlated with mortality, and were risk factors in PDAC. Both Nectin-2 and DDX3 are independent prognostic factors (Tables 4 and 5).

## 4. Discussion

The expression of Nectin-2 and DDX3 in PDAC has not been previously reported, although their expressions have been associated with the progression and prognosis of a variety of tumors. This study investigated Nectin-2 and DDX3 protein expression in PDAC tumors, peritumoral tissues, benign pancreatic lesions, and normal pancreatic tissues using immunohistochemistry. A significant increase in Nectin-2 and DDX3 expression in PDAC tumors was observed. Positive Nectin-2 and DDX3 expressions are associated with poor differentiation, invasion, metastasis, and poor prognosis of PDAC.

Nectin-2 is located at the connections between endothelial cells, and the interactions between these two subtypes transfer extracellular signaling into cells [30]. Takahashi et al. [31] study found that Nectin-2 plays important roles in cell adhesion, migration, and polarization [6]. These functions of Nectin-2 implicate its possible roles in tumor cell survival and proliferation. Indeed, a previous study using a polyclonal antibody specific to Nectin-2 showed that Nectin-2 is involved in the proliferation of ovarian cancer cells [32]. Moreover, recent studies have demonstrated that Nectin-2 is highly expressed in epithelial malignancies and correlates with high malignancy, fast progression, and/or poor prognosis of human breast cancer [14, 15], ovarian cancer [15], gallbladder cancer [16], and the invasive squamous carcinomas of the human uterine cervix [17]. However, the clinical significance of Nectin-2 has not yet been addressed in PDAC. This study first demonstrated that positive Nectin-2 expression significantly correlated with clinical progression, as indicated by large tumor size, lymph node metastasis, and tumor cell invasion. The observed association between Nectin-2 positive expression and the progression of PDAC may be associated with its functions in cell proliferation, migration, and signaling transduction. Moreover, positive Nectin-2 expression correlated with shorter survival. Cox multivariate analysis suggested that positive Nectin-2 expression is an independent prognostic factor associated with poor prognosis in PDAC patients.

Human DDX3 plays an important role in gene expression and the translation process. DDX3 is also involved in cell cycle regulation and cell apoptosis [33]. DDX3 promotes p21WAF1/CIP1 promoter activity and subsequent gene expression, which can inhibit tumor growth, making DDX3 a tumor suppressor gene [34]. However, reports on the roles of DDX3 in various cancers are controversial. For example, elevated DDX3 expression has been observed in tumor tissues of breast cancer [24], non-small cell lung cancer [25, 26], oral squamous cell carcinoma [27], and gallbladder cancer [16]. DDX3 is also found to be involved in carcinogenesis through enhancing cellular proliferation and maintaining genomic integrity [35]. A recent study found low/negative DDX3 expression in tumor cells of oral squamous cell carcinoma, which was significantly associated with aggressive clinical manifestations, and DDX3 is an independent survival predictor in nonsmoker patients with oral squamous cell carcinoma [27]. In contrast, high DDX3 expression is closely correlated with progression and poor prognosis of gallbladder cancer [16]. Therefore, the current findings suggest that the role of DDX3 in cancer is tumor type specific. In this study, DDX3 protein expression was significantly increased in PDAC tumors. This is the first study that reported DDX3 expression in PDAC. Moreover, positive DDX3 expression was significantly related to poor differentiation of the tumor and severe clinical manifestations. Positive DDX3 expression also correlated with shorter survival and poor prognosis in PDAC patients.

## 5. Conclusions

This study suggests that Nectin-2 and DDX3 are involved in the progression of PDAC, and positive Nectin-2 and DDX3 expression were associated with shorter survival and poor prognosis in patients with PDAC. Positive Nectin-2 and DDX3 expression could be diagnostic markers of PDAC.

## Figures and Tables

**Table 1:** Comparison of Nectin-2 and DDX3 expression in normal, benign, and malignant pancreatic tissues.

Tissue types	Case number	Nectin-2 positive (%)	DDX3 positive (%)
Pancreatic ductal adenocarcinoma	106	58 (54.7)	55 (51.9)
Peritumoral tissues	35	9 (25.7)^*∗∗*^	8 (22.9)^*∗∗*^
Benign tissues	55	10 (18.2)^*∗∗*^	11 (20.0)^*∗∗*^
Normal pancreatic tissues	13	0 (0.0)^*∗∗*^	0 (0.0)^*∗∗*^

Compared to pancreatic ductal adenocarcinoma, ^*∗∗*^ *P* < 0.01.

**Table 2:** Correlations of Nectin-2 and DDX3 protein expression with the clinicopathological characteristics of pancreatic ductal adenocarcinoma.

CPC	Case number	Nectin-2			DDX3		
		Positive number (%)	*χ* ^2^	*P* value	Positive number (%)	*χ* ^2^	*P* value
Age (year)							
≤45	22	12 (54.5)	0.000	0.986	14 (63.6)	1.535	0.215
>45	84	46 (54.8)			41 (48.8)		
Sex							
Male	61	33 (54.1)	0.022	0.882	31 (50.8)	0.066	0.798
Female	45	25 (55.6)			24 (53.3)		
Differentiation							
Well	38	16 (42.1)	4.424	0.109	14 (36.8)	9.341	0.009
Moderately	35	20 (57.1)			17 (48.6)		
Poorly	33	22 (66.7)			24 (72.7)		
Tumor size							
≤3 cm	13	5 (38.5)	2.258	0.323	6 (46.2)	0.345	0.841
3–5 cm	68	37 (54.5)			35 (51.5)		
>5 cm	25	16 (64.0)			14 (56.0)		
Lymph node metastasis							
No	77	34 (44.2)	12.670	0.000	35 (45.5)	4.664	0.031
Yes	29	24 (82.8)			20 (69.0)		
Invasion							
No	42	14 (33.3)	12.838	0.000	13 (31.0)	12.212	0.000
Yes	64	44 (68.8)			42 (65.6)		
TNM stage							
T1	11	4 (36.4)	13.190	0.004	3 (27.3)	14.848	0.002
T2	42	16 (38.1)			16 (38.1)		
T3	37	25 (67.6)			22 (59.5)		
T4	16	13 (81.3)			14 (87.5)		

CPC: clinical and pathological characteristics.

**Table 3:** Correlations of clinicopathological characteristics and Nectin-2 and DDX3 expression with mean survival in patients with pancreatic ductal adenocarcinoma.

Groups	Case number (*n*)	Mean survival (median) month	*χ* ^2^	*P* value
Sex				
Male	61	9.98 (13)	1.656	0.198
Female	45	8.61 (11.5)		
Age (year)				
≤45	22	8.18 (11)	2.144	0.143
>45	84	9.73 (13)		
Differentiation				
Well	38	11.27 (13.5)	17.786	0.000
Moderately	35	9.74 (12)		
Poorly	33	6.86 (8)		
Tumor size				
<3 cm	13	13.46 (13)	7.504	0.023
3~5 cm	68	9.34 (12)		
>5 cm	25	7.40 (13.5)		
TNM stage				
I	11	16.46 (17.5)	80.807	0.000
II	42	11.37 (12.5)		
III	37	7.14 (9.5)		
IV	16	4.56 (5)		
Lymph node metastasis				
No	77	10.64 (13)	27.120	0.000
Yes	29	6.35 (7)		
Invasion				
No	42	13.33 (14.5)	46.949	0.000
Yes	54	6.83 (9.5)		
Nectin-2				
−	48	12.63 (13.5)	44.074	0.000
+	56	6.73 (8.5)		
DDX3				
−	55	12.05 (14.5)	28.608	0.000
+	51	6.98 (10)		

**Table 4:** Multivariate Cox regression analysis of survival rate in patients with pancreatic ductal adenocarcinoma.

Group	Factors	*B*	SE	Wald	*P*	RR	95% CI	
							Lower	Upper
Differentiation	Well/moderately/poorly	1.287	0.466	7.63	0.006	3.62	1.45	9.03
Tumor size	<3 cm/3–5 cm/>5 cm	1.589	0.627	6.42	0.011	4.90	1.43	16.04
Lymph node metastasis	no/yes	2.574	0.782	10.83	0.001	13.12	2.83	60.75
Invasion	no/yes	2.693	0.801	11.30	0.001	14.78	3.07	71.02
TNM stage	I/II/III/IV	1.485	0.537	7.65	0.006	4.41	1.54	12.65
Nectin-2	−/+	3.016	0.865	12.16	0.000	20.41	3.75	111.21

CI: confidence interval.

**Table 5:** Multivariate Cox regression analysis of survival rate in patients with pancreatic ductal adenocarcinoma.

Group	Factors	*B*	SE	Wald	*P*	RR	95% CI	
							Lower	Upper
Differentiation	Well/moderately/poorly	1.126	0.409	7.58	0.006	3.08	1.38	6.87
Tumor size	<3 cm/3–5 cm/>5 cm	1.633	0.661	6.10	0.013	5.12	1.40	18.70
Lymph node metastasis	No/yes	2.785	0.822	11.48	0.001	16.20	3.23	81.14
Invasion	No/yes	2.874	0.817	12.37	0.000	17.71	3.57	87.82
TNM stage	I/II/III/IV	1.762	0.628	7.87	0.005	5.82	1.70	19.9
DDX3	−/+	2.141	0.674	10.9	0.001	8.51	2.27	31.88

CI: confidence interval.
